# Clinical outcomes and midterm durability of the preloading coil-in-plug technique in various arterial territories

**DOI:** 10.1186/s42155-026-00790-9

**Published:** 2026-10-30

**Authors:** Takahiro Nakai, Shinichi Iwakoshi, Mako Iwata, Hayato Kishida, Keisuke Oshima, Shigeo Ichihashi, Toshihiro Tanaka

**Affiliations:** Department of Radiology, Nara Medical University, Kashihara, Japan

**Keywords:** Amplatzer vascular plug, Coil-in-plug, Arterial embolization, Vascular plug, Coil embolization, Recanalization

## Abstract

**Background:**

Conventional coil embolization of large or high-flow arteries may require multiple coils and a long embolization segment. The Amplatzer Vascular Plug (AVP) enables controlled deployment and repositioning; however, complete occlusion partly depends on thrombus formation, and residual flow or recanalization may occur. The preloading coil-in-plug (P-CIP) technique combines the positional stability of an AVP with internal coil packing. This study evaluated its technical feasibility, procedural characteristics, safety, and mid-term imaging outcomes across different arterial territories.

**Methods:**

This retrospective single-center study evaluated consecutive patients who underwent P-CIP embolization between April 2018 and April 2026. Before insertion, a microcatheter was passed through the nitinol mesh into the internal space of an AVP I outside the patient. The plug and microcatheter were advanced together and deployed at the target site before plug detachment. Coils were then packed within the expanded plug through the preloaded microcatheter. Technical success, AVP-to-vessel diameter ratio, coil use, coil-packing time, procedure-related complications, target-vessel recanalization, and reintervention were evaluated. Imaging follow-up was based on contrast-enhanced computed tomography.

**Results:**

The study included 34 patients, of whom 21 were men, with 40 embolized vessels. The target vessels were the celiac artery in 6 procedures, internal iliac artery in 25, superior or inferior gluteal artery in 8, and external iliac artery in 1. Technical success and final angiographic occlusion were achieved in all 40 vessels. The mean AVP-to-vessel diameter ratio was 1.44, corresponding to 44% oversizing. The mean number of coils used per AVP was 3.13, and the mean coil-packing time was 10.7 min. During a mean imaging follow-up of 619 days, no procedure-related complications, target-vessel recanalization, device migration, or reintervention occurred. Ten patients died during follow-up, but none of the deaths was related to embolization.

**Conclusions:**

P-CIP embolization was technically feasible across multiple arterial territories and achieved sustained target-vessel occlusion during mid-term imaging follow-up. The technique may be useful when controlled short-segment embolization is required. Comparative studies are needed to determine whether P-CIP provides advantages over conventional coil embolization or AVP placement alone.

## Introduction

Transcatheter arterial embolization is widely used for various clinical settings. However, conventional coil embolization of large or high-flow vessels may require multiple coils and a relatively long embolization segment. In addition, insufficient coil stability may result in coil migration, incomplete vessel occlusion, or recanalization.

The Amplatzer Vascular Plug (AVP; Abbott, Plymouth, MN, USA) enables controlled deployment and repositioning and is widely used for embolization of relatively large vessels. However, complete occlusion after AVP placement depends partly on thrombus formation and may not be immediate. Persistent flow through or around the plug may occur, particularly in high-flow vessels or vessels with irregular luminal morphology. Recanalization after embolization with the single-segment AVP has also been reported [1, 2], although robust comparative clinical evidence regarding differences among AVP models remains limited.

To address this limitation of AVP, the coil-in-plug (CIP) technique was developed, combining the positional stability of the AVP with the embolic effects of metallic coils. In the original CIP technique, a microcatheter is advanced through the nitinol mesh into an AVP after the plug has been deployed within the target vessel, and coils are subsequently packed into the expanded plug before detachment [3]. However, cannulation of the deployed plug may be technically challenging. To simplify this step, the preloading CIP technique was subsequently introduced, in which a microcatheter is inserted into the AVP outside the patient before the plug and microcatheter are advanced together to the target vessel [4, 5]. The AVP acts as a scaffold that limits coil migration, while internal coil packing increases the amount of embolic material and reduces residual flow channels through the nitinol mesh.

Previous studies have demonstrated the feasibility of CIP for embolization of the internal iliac artery, left subclavian artery, and large portosystemic shunts [6–8]. However, most reports have been technical notes, case reports, or small studies limited to a single vascular territory. Data regarding the use of CIP across different arterial territories and its mid-term durability remain limited.

The purpose of this study was to evaluate the technical feasibility, procedural characteristics, safety, and midterm imaging outcomes of the P-CIP technique in various arterial territories.

## Materials and methods

### Study design and patients

This retrospective observational study was approved by the institutional review board, which waived the requirement for individual written informed consent because of the retrospective study design. All patients had provided written informed consent for the endovascular procedure.

The institutional interventional radiology database was reviewed to identify consecutive patients who underwent arterial embolization using the P-CIP technique between April 2018 and April 2026.

Patients were included when an AVP I was placed in a target artery and one or more metallic coils were intentionally deployed within the plug before AVP detachment.

### Preloading coil-in-plug technique

Before insertion of the AVP, an AVP was prepared using the preloading coil-in-plug technique. A microcatheter was passed through the nitinol mesh into the internal space of the AVP outside the patient, and the plug and microcatheter were then loaded together into an appropriately sized guiding sheath or guiding catheter.

**Fig. 1:**
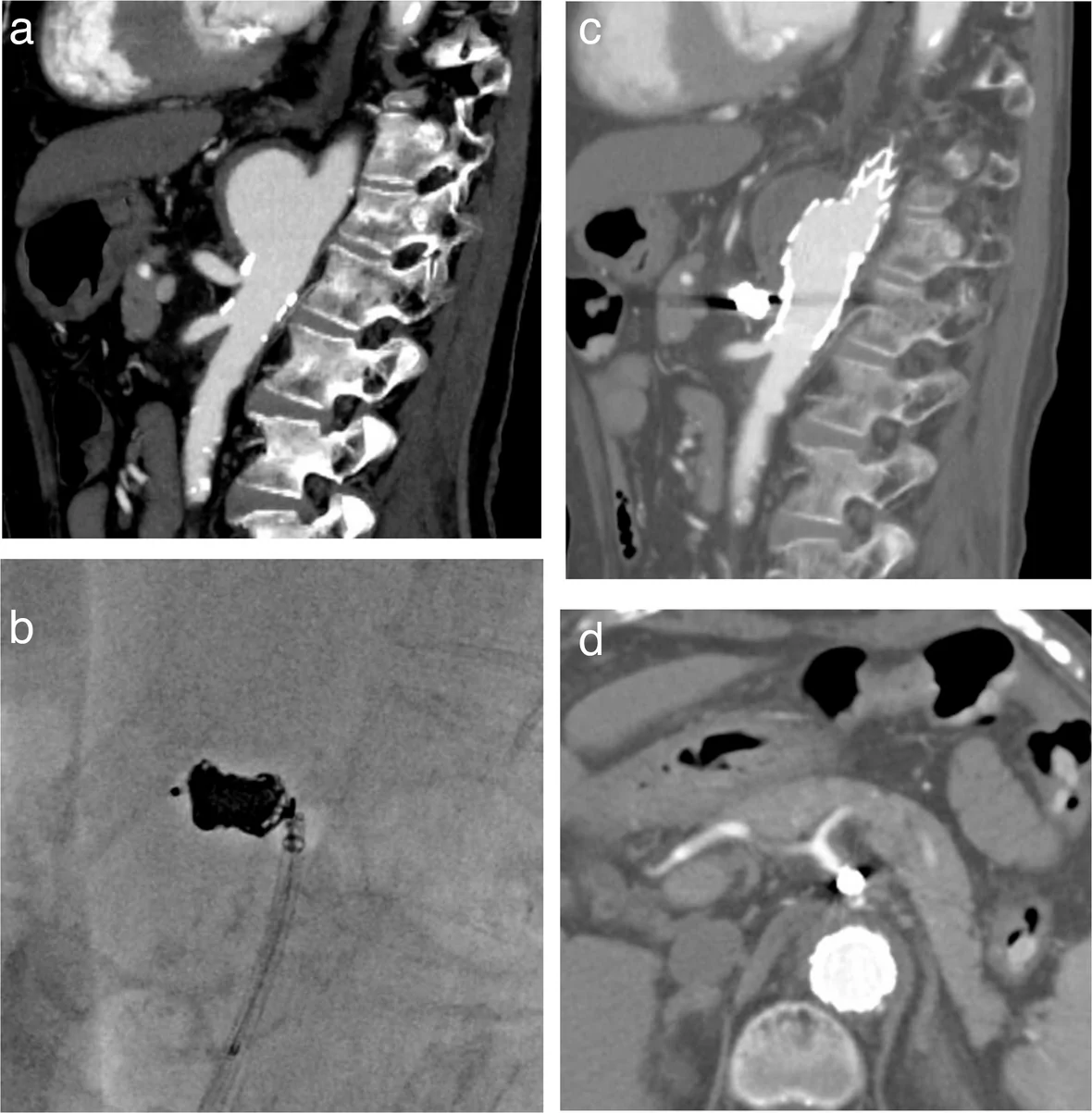
Representative case of celiac artery embolization using the preloading coil-in-plug technique. A 79-year-old woman presented with a saccular aneurysm at the thoracoabdominal aorta. **a** Preoperative sagittal contrast-enhanced CT shows the saccular aneurysm immediately proximal to the origin of the celiac artery. Celiac artery embolization was planned to extend the distal landing zone for stent-graft placement. **b** A fluoroscopic image obtained during preloading coil-in-plug embolization shows a 10-mm Amplatzer Vascular Plug I deployed in the celiac trunk and packed with POD Packing Coils (Penumbra, Alameda, CA, USA). All coils are confined within the plug. **c** Sagittal contrast-enhanced CT obtained 1 week after TEVAR shows complete exclusion of the aneurysm without any endoleak, which indicated the occlusion of the celiac trunk. **d** Postoperative CT angiography demonstrates preserved opacification of the common hepatic and splenic arteries despite celiac trunk embolization

The guiding sheath or catheter was advanced near the target vessel, and the preloaded AVP was deployed at the intended embolization site without immediate detachment. After confirming an appropriate device position, detachable coils were delivered through the preloaded microcatheter and packed within the expanded AVP. The AVP size was generally approximately 30–50% larger than the measured target-vessel diameter. Coils were generally selected with a nominal diameter approximately equal to the target-vessel diameter. The AVP was detached after confirming satisfactory device position, absence of clinically significant coil protrusion, and adequate reduction or cessation of target-vessel flow. Representative procedural images illustrating the preloading coil-in-plug technique are shown in Fig. 1.

### Outcomes measured

The target-vessel diameter was assessed on the preoperative contrast-enhanced computed tomography. The target-vessel location and diameter, nominal AVP diameter, oversizing ratio, number of coils, and coil-packing time were recorded for each vessel. Coil-packing time was defined as the interval from the procedural time point immediately before or immediately after AVP deployment, depending on the available angiographic record, to the angiographic acquisition performed immediately before AVP detachment.

### Follow-up imaging

Follow-up contrast-enhanced CT examinations were generally performed as part of routine postprocedural surveillance at approximately 1 week, 6 months, and 1 year after treatment, and every 6–12 months thereafter. The exact timing of follow-up imaging varied according to the patient’s clinical condition. Follow-up images were evaluated for target-vessel recanalization, residual flow through or around the plug, device migration, and ischemic complications. The imaging follow-up period was calculated from the date of embolization to the most recent contrast-enhanced computed tomography examination in which the treated vessel could be evaluated.

### Definitions

Technical success was defined as successful placement of the AVP at the intended site, successful deployment of coils within the plug, and satisfactory angiographic occlusion of the target vessel at the end of the procedure.

Satisfactory angiographic occlusion was defined as complete cessation or marked reduction of antegrade flow through the target vessel on final angiography.

Procedure-related complications included non-target embolization, device migration, arterial dissection or rupture, clinically significant ischemia, access-site complications requiring treatment, and other adverse events considered related to the embolization procedure.

Recanalization was defined as contrast opacification through or around the embolized segment on follow-up contrast-enhanced computed tomography. Reintervention was defined as any additional endovascular or surgical treatment performed because of persistent flow, recanalization, or an embolization-related complication.

### Statistical analysis

Continuous variables are presented as mean ± standard deviation or median with interquartile range, as appropriate. Categorical variables are presented as numbers and percentages.

The analysis was performed primarily on a target-vessel basis. Because this study was descriptive and did not include a control group, no multivariable regression or time-to-event analysis was planned. Statistical analyses were conducted using IBM SPSS Statistics, version 27.0.1.0 (IBM Corp., Armonk, NY, USA).

## Results

### Patient and target-vessel characteristics

Thirty-four patients were included, 21 being men (61.8%). The mean age was 77.3 years. The baseline characteristics and indications for embolization are summarized in Table 1.

**Table 1:** Baseline characteristics

	Mean ± SD, or *n* (%)
Age, years	77.7 ± 9.2
Sex	
Male	21/34 (61.8%)
Female	13/34 (38.2%)
Smoking status	
Never	19/34 (55.9%)
Previous	11/34 (32.4%)
Current	4/34 (11.7%)
Hypertension	33/34 (97.1%)
Diabetes	3/34 (8.8%)
Dyslipidemia	12/34 (35.3%)
Chronic kidney disease (eGFR < 45)	6/34 (17.6%)
Dialysis	2/34 (5.9%)
Previous coronary disease	2/34 (5.9%)
Previous cerebrovascular disease	5/34 (14.7%)
Pre-operative medication	
Platelet-inhibitor/anticoagulant	11/34 (32.4%)
Statin	12/34 (35.3%)
Pre-operative platelet count, *10^4/dL	20.8 ± 7.1
Pre-operative fibrinogen, mg/dL	369 ± 161
	*n* (%)
Number of target vessels	
1	29/34 (85.3%)
2	4/34 (11.8%) bilateral proximal IIA 2 distal IIA + contralateral SGA 1 SGA + ipsilateral IGA 1
3	1/34 (2.9%) Bilateral IGA + SGA 1
Target of embolization	
Celiac artery (CA)	6/40 (15.0%)
Proximal internal iliac artery (pIIA)	22/40 (55.0%)
Distal internal iliac artery (dIIA)	3/40 (7.5%)
Superior/inferior gluteal artery (SGA/IGA)	8/40 (20.0%)
External iliac artery (EIA)	1/40 (2.5%)
Indication of embolization–CA	
Shortage of landing zone	4/6 (66.7%)
Stagnation of pseudolumen flow of IMH	2/6 (33.3%)
Indication of embolization–pIIA	
Shortage of distal landing	10/22 (45.5%)
Concomitant of CIA aneurysm	12/22 (54.5%)
Indication of embolization–dIIA/SGA/IGA	
Concomitant of IIA aneurysm	11/11 (100%)
Indication of embolization–EIA	
Exclusion of anastomotic leakage of EIA-femoral artery bypass	1/1 (100%)

A total of 40 target vessels were treated. Twenty-nine patients underwent embolization of one vessel, 4 underwent embolization of two vessels, and 1 underwent embolization of three vessels.

The target vessels were the celiac artery in 6 cases (15.0%), proximal internal iliac artery in 22 (55.0%), distal internal iliac artery in 3 (7.5%), superior or inferior gluteal artery in 8 (20.0%), and external iliac artery in 1 (2.5%). All target vessels in this cohort were treated in conjunction with proximal stentgraft implantation. Twenty-nine vessels had the adjacent-stentgraft configuration (celiac artery, proximal internal iliac artery, external iliac artery), whereas 11 had the aneurysm-interposed configuration (distal internal iliac artery, superior or inferior gluteal artery). In the latter configuration, an internal iliac artery aneurysm sac was interposed between the proximal stentgraft and the P-CIP-treated branch. Because these configurations have distinct hemodynamic characteristics, follow-up imaging outcomes were summarized separately. The mean target-vessel diameter was 7.7 mm.

### Technical results

P-CIP technical success was achieved in all 40 target vessels (100%). Satisfactory final angiographic occlusion was achieved in all vessels.

Concomitant embolization of adjacent branches outside the AVP-covered segment was performed in 10 vessels.

The mean AVP oversizing ratio was 144 ± 20%. The distribution of AVP models and sizes is shown in Table 2.

**Table 2:** Distribution of AVP models

Diameter of AVP, mm	Overall *n* = 40	CA *n *= 6	IIA *n* = 25	SGA/IGA *n* = 8	EIA *n* = 1
6	1 (2.5%)	0 (0%)	0 (0%)	1 (12.5%)	0 (0%)
8	8 (20.0%)	1 (16.7%)	4 (16.0%)	3 (37.5%)	0 (0%)
10	9 (22.5%)	2 (33.3%)	4 (16.0%)	3 (37.5%)	0 (0%)
12	17 (42.5%)	2 (33.3%)	13 (52.0%)	1 (12.5%)	1 (100%)
14	4 (10.0%)	1 (16.7%)	3 (12.0%)	0 (0%)	0 (0%)
16	1 (2.5%)	0 (0%)	1 (4.0%)	0 (0%)	0 (0%)

Data are *n *(%)

### Coil use and coil-packing time

Table 3 summarizes the microcatheters and coils used, including catheter size, coil type and manufacturer, coil diameter and length, and the number of coils deployed within each AVP.

**Table 3:** Microcatheters and embolization coils used in the preloaded coil-in-plug technique

Preloaded microcatheters (distal tip diameter, manufacturer)	
Breakthrough 2 marker (1.9 F, Boston Scientific, Marlborough, MA, USA)	10
LIGHTHOUSE (2.0 F, Piolax medical device, Kanagawa, Japan)	1
Progreat β3 2 marker (2.2 Fr, Terumo, Tokyo, Japan)	10
Progreat 2.4 Fr 2 marker (2.4 Fr, Terumo, Tokyo, Japan)	1
Carry leon Highflow (2.6 F, UTM, Aichi, Japan)	2
Lantern (2.6 F, Penumbra, Alameda, CA, USA)	15
Progreat Ω 2 marker (2.8 Fr, Terumo, Tokyo, Japan)	1
AVP sizes and coils used for packing	
Packed coil–AVP 6 mm (*n* = 1)	
POD System (Penumbra, Alameda, CA, USA)	
Packing coil 30 cm	1
Packed coils–AVP 8 mm (*n* = 8)	
AZUR 18 (Terumo, Tokyo, Japan)	
4 mm × 10 cm	1
6 mm × 20 cm	2
EMBOLD Fibered Coil (Boston Scientific, Marlborough, MA, USA)	
5 mm × 15 cm	1
6 mm × 20 cm	2
Interlock-18 Fibered IDC Occlusion System (Boston Scientific, Marlborough, MA, USA)	
6 mm × 20 cm	3
8 mm × 20 cm	3
POD System (Penumbra, Alameda, CA, USA)	
Packing coil 15 cm	2
Packing coil 30 cm	2
Packing coil 45 cm	1
Packing coil 60 cm	2
Target XXL 360 Coils (Stryler, Kalamazoo, MI, USA)	
6 mm × 40 cm	1
Packed coils–AVP 10 mm (*n* = 9)	
AZUR Soft 3D (Terumo, Tokyo, Japan)	
7 mm × 28 cm	3
EMBOLD Fibered Coil (Boston Scientific, Marlborough, MA, USA)	
4 mm × 8 cm	2
8 mm × 20 cm	2
i-ED COIL (Kaneka medics, Osaka, Japan)	
Complex Infini 5 mm × 20 cm	1
Infini 14 mm × 50 cm	1
Nester Embolization Coil .018inch (Cook Medical, Bloomington, IN, USA)	
8 mm × 14 cm	6
POD System (Penumbra, Alameda, CA, USA)	
Packing coil 15 cm	2
Packing coil 45 cm	2
Packing coil 60 cm	2
Packed coils–AVP 12 mm (*n* = 17)	
AZUR CX18 (Terumo, Tokyo, Japan)	
7 mm × 24 cm	1
8 mm × 28 cm	4
10 mm × 32 cm	2
12 mm × 38 cm	1
EMBOLD Fibered Coil (Boston Scientific, Marlborough, MA, USA)	
6 mm × 20 cm	3
8 mm × 20 cm	7
10 mm × 50 cm	2
i-ED COIL (Kaneka medics, Osaka, Japan)	
Infini 14 mm × 15 cm	2
Infini 14 mm × 30 cm	3
Infini 14 mm × 50 cm	4
Interlock-18 Fibered IDC Occlusion System (Boston Scientific, Marlborough, MA, USA)	
6 mm × 20 cm	3
8 mm × 20 cm	4
10 mm × 30 cm	2
12 mm × 30 cm	3
Avenir Coil System (Wallaby Medical, Laguna Hills, CA, USA)	
MEGAnir Helical 5 mm × 20 cm	2
MEGAnir Helical 6 mm × 30 cm	3
MEGAnir Helical 8 mm × 30 cm	4
POD System (Penumbra, Alameda, CA, USA)	
Packing coil 45 cm	1
Packing coil 60 cm	8
POD8 60 cm	1
Ruby Coil System (Penumbra, Alameda, CA, USA)	
Standard 12 mm × 60 cm	1
Packed coils–AVP 14 mm (*n* = 4)	
Allure-CS Coil System (Century Medical, Tokyo, Japan)	
Complex18 8 mm × 27 cm	3
Complex18 10 mm × 34 cm	1
Interlock-18 Fibered IDC Occlusion System (Boston Scientific, Marlborough, MA, USA)	
8 mm × 20 cm	1
10 mm × 30 cm	3
10 mm × 50 cm	2
12 mm × 30 cm	1
14mm × 30 cm	2
18mm × 50 cm	2
POD System (Penumbra, Alameda, CA, USA)	
Packing coil 30 cm	1
Packing coil 60 cm	2
Ruby Coil System (Penumbra, Alameda, CA, USA)	
Soft 10mm × 35 cm	2
Standard 12 mm × 60 cm	1
Packed coils–AVP 16mm (*n* = 1)	
POD System (Penumbra, Alameda, CA, USA)	
Packing coil 60 cm	3

The mean number of coils packed within each AVP was 3.13 ± 1.76,

The mean coil-packing time was 10.7 ± 2.1 min.

### Complications and clinical outcomes

No procedure-related complication occurred during the procedure or follow-up. Specifically, there was no non-target embolization, clinically significant ischemia, device migration, vessel injury, or embolization-related reintervention.

Ten patients died during the observation period. All 10 had undergone single-vessel embolization. None of the deaths was related to the embolization procedure or to ischemia of the embolized vascular territory. Causes of death were cancer (*n* = 4), pneumonia (*n* = 2), cardiac death (*n* = 1), chronic renal failure (*n* = 1), retroperitoneal bleeding (*n* = 1), and sepsis (*n* = 1).

### Imaging follow-up

The mean imaging follow-up period from embolization to the most recent contrast-enhanced computed tomography examination was 619 ± 587 days (median 367 days, IQR 165–930 days; range 3–1826 days).

No target-vessel recanalization, persistent flow requiring treatment, or device migration was observed in either the adjacent-stentgraft configuration (0/29 vessels) or the aneurysm-interposed configuration (0/11 vessels) during the available imaging follow-up. No patient required repeat embolization or surgical treatment of a target vessel.

For the 10 patients who died during follow-up, the final available contrast-enhanced computed tomography examination before death demonstrated maintained target-vessel occlusion in all 10 cases.

## Discussion

This study investigated the mid-term outcome of preloading coil-in-plug (P-CIP) technique in various arterial territories. P-CIP was technically successful in all 40 target vessels, and no recanalization, procedure-related complication, or reintervention occurred during a mean imaging follow-up of 619 days. The technique was applied to the celiac, internal iliac, gluteal, and external iliac arteries. These findings support the technical feasibility of P-CIP across different arterial territories and provide observational mid-term data regarding maintenance of occlusion at the treated segment.

The CIP technique combines the advantages of an AVP I and metallic coils. The AVP I provided a stable framework that can be accurately positioned and repositioned before detachment. Metallic coils packed within the plug increase the amount of thrombogenic material and reduce residual channels through the nitinol mesh. The plug also reduces the risk of distal coil migration, particularly in relatively large or high-flow vessels.

Previous studies of CIP techniques have primarily focused on internal iliac artery embolization during endovascular aortic aneurysm repair [3, 4, 8]. Early reports demonstrated that CIP could achieve ultrashort- or short-segment embolization, and a small comparative study showed that conventional CIP required fewer coils and resulted in a shorter embolization length than conventional coil embolization [3, 4]. More recently, a comparative study of the preloading CIP technique demonstrated fewer coils, a higher immediate complete occlusion rate, and a shorter embolization range than conventional coil embolization [8].

The present study included 40 target vessels and represents a relatively large clinical series of CIP-based embolization. Moreover, unlike previous studies limited to a single vascular territory, the present cohort included the celiac, internal iliac, gluteal, and external iliac arteries. The successful application of the technique across these arterial territories suggests that CIP may be adaptable to vessels with different diameters, configurations, and hemodynamic conditions. Importantly, all target vessels were treated in conjunction with proximal stentgraft implantation. The adjacent-stentgraft configuration and aneurysm-interposed configuration are hemodynamically distinct. In the latter, proximal inflow was modified by stentgraft coverage and an internal iliac artery aneurysm sac was interposed between the stentgraft and the P-CIP-treated distal branch. Therefore, maintenance of distal branch occlusion in these cases cannot be attributed to P-CIP alone. The stratified findings should be interpreted as imaging outcomes of the combined treatment configuration rather than as a direct comparison of intrinsic P-CIP performance across vascular territories, although the heterogeneous anatomy and concomitant stentgraft implantation limit direct comparisons among vascular territories.

The mean number of coils packed within each AVP was 3.1, which was comparable to the mean values of 3.8 and 3.36 coils reported in previous studies of conventional and preloading CIP techniques, respectively [4, 8].

In the present study, the mean oversizing ratio was approximately 140%, which was within the generally recommended range of 30–50% oversizing for AVP embolization [9]. These findings suggest that the conventional sizing strategy used for AVP placement may also be appropriate when performing the CIP technique.

The mean coil-packing time was approximately 10.7 min. The starting time point was the procedural record immediately before or after AVP I deployment, depending on the available angiographic sequence, and the endpoint was the angiographic assessment performed immediately before plug detachment. Although this value cannot be directly compared with the total embolization times reported in previous studies, it suggests that internal coil packing can generally be completed within a clinically acceptable period.

No CT-detectable recanalization was observed during the available imaging follow-up. Although this observation is clinically encouraging, it should be interpreted cautiously. With zero events among 40 vessels, the rule of three gives an approximate upper 95% confidence bound of 7.5% for the underlying recanalization rate. Therefore, the true event rate cannot be assumed to be zero. AVP occlusion relies partly on thrombus formation, and persistent or recurrent flow may occur when the device does not completely conform to the vessel wall or when thrombosis is delayed. Internal coil packing creates an additional mechanical and thrombogenic barrier within the plug and may reduce dependence on thrombus formation alone. Nevertheless, the present study did not include an AVP alone or conventional coil control group. Therefore, it cannot establish that P-CIP reduces recanalization or improves procedural efficiency compared with other embolization techniques.

More recently, vascular occlusion devices have expanded the options for short-segment embolization. Shape-memory polymer-based devices, including IMPEDE and IMPEDE-FX Embolization Plugs (Shape Memory Medical, San Jose, CA, USA), use a porous polymer scaffold that expands in vivo and contains relatively little radiopaque metallic material [10]. Low-profile membrane-covered devices, including Micro Vascular Plug (Medtronic, Minneapolis, MN, USA) and AZUR Vascular Plug (MicroVention, Aliso Viejo, CA, USA), provide alternative approaches to focal vessel occlusion [11, 12]. Direct comparative data between these devices and P-CIP remain limited.

This study had several limitations: first, this was a retrospective, single-center study without a control group. Therefore, the relative advantages of CIP over AVP alone or conventional coil embolization could therefore not be determined. Second, the target vessels and clinical indications were heterogeneous. Differences in vessel diameter, blood flow, morphology, and collateral circulation may have influenced outcomes. Third, five patients contributed more than one target vessel, and the vessel-level observations were not completely independent. Fourth, imaging follow-up duration varied, and death shortened the available surveillance period in 10 patients. Fifth, metallic coils can produce CT artifacts that may obscure subtle residual or recurrent contrast opacification and may therefore reduce the sensitivity of CT-based assessment compared with AVP-alone embolization [13]. Sixth, although no device migration or other device-related mechanical complication was observed during the study period, rare late complications cannot be excluded. Delayed mechanical complications, including erosion of vascular plugs into adjacent structures, have been reported [14], and longer-term surveillance is required to further assess mechanical safety. Finally, immediate angiographic occlusion was not evaluated using a prospectively defined grading system. Future studies should use standardized angiographic criteria and assess whether residual flow immediately after AVP I detachment is associated with subsequent recanalization.

The present findings suggest that P-CIP can be applied to arteries with different anatomical and hemodynamic characteristics while maintaining durable vessel occlusion. The combination of the positional stability of the AVP I and internal coil packing may be particularly advantageous when occlusion within a short embolization segment is required. Further comparative studies are needed to clarify the optimal degree of plug oversizing and coil packing and to determine whether this technique offers advantages over conventional coil embolization or AVP placement alone.

## Conclusion

The P-CIP technique was successfully performed in various arterial territories, with no CT-detectable recanalization or reintervention observed during mid-term imaging follow-up. This technique appears to be a feasible option for controlled short-segment arterial embolization.

## Data Availability

The datasets generated and/or analyzed during the current study are available from the corresponding author on reasonable request.
